# Identification of accessory olfactory system and medial amygdala in the zebrafish

**DOI:** 10.1038/srep44295

**Published:** 2017-03-14

**Authors:** Daniela Biechl, Kristin Tietje, Soojin Ryu, Benedikt Grothe, Gabriele Gerlach, Mario F. Wullimann

**Affiliations:** 1 Graduate School of Systemic Neurosciences & Department Biology II, Ludwig-Maximilians-Universität Munich, Grosshadernerstr. 2, 82152 Planegg-Martinsried, Germany; 2 Department of Biology and Environmental Sciences, Carl von Ossietzky University Oldenburg, Carl von Ossietzky Str. 9-11, 26111 Oldenburg, Germany; 3 Focus Program Translational Neuroscience, University Medical Center, Johannes Gutenberg University Mainz, Langenbeckstr. 1, 55131 Mainz, Germany

## Abstract

Zebrafish larvae imprint on visual and olfactory cues of their kin on day 5 and 6 postfertilization, respectively. Only imprinted (but not non-imprinted) larvae show strongly activated crypt (and some microvillous) cells demonstrated by pERK levels after subsequent exposure to kin odor. Here, we investigate the olfactory bulb of zebrafish larvae for activated neurons located at the sole glomerulus mdG2 which receives crypt cell input. Imprinted larvae show a significantly increased activation of olfactory bulb cells compared to non-imprinted larvae after exposure to kin odor. Surprisingly, pERK activated Orthopedia-positive cell numbers in the intermediate ventral telencephalic nucleus were higher in non-imprinted, kin odor stimulated larvae compared to control and to kin-odor stimulated imprinted larvae and control. Moreover, DiI tracing experiments in adult zebrafish show a neuronal circuit from crypt/microvillous olfactory sensory neurons via dorsomedial olfactory bulb and intermediate ventral telencephalic nucleus (thus, arguably the teleostean medial amygdala) to tuberal hypothalamus, demonstrating for the first time an accessory olfactory system in teleosts.

Bony and cartilaginous fishes have a single main olfactory epithelium (MOE). Thus, in contrast to most tetrapods, they lack an additional vomeronasal organ^1^,^2^,^3^. Nevertheless, teleost fishes have in addition to ciliated olfactory sensory neurons, which are typical for the tetrapod main olfactory epithelium, also microvillous olfactory sensory neurons, which in tetrapods reside in the vomeronasal organ. These fish microvillous olfactory sensory neurons are intermingled with ciliated olfactory sensory neurons within the unitary main olfactory epithelium^4^,^5^. The presence of two olfactory sensory neuron types matches with receptor class and associated G-proteins reported in mammals. Teleost olfactory receptors (ORs) and trace amine receptors (TAARs) associated with Gαolf are located on the cilia of ciliated olfactory sensory neurons, while V1Rs (ora) associated with Gαi, and V2Rs (OlfC) associated with Gαo are present on microvilli of microvillous olfactory sensory neurons both in mammals and teleosts^3^,^6^,^7^. However, in goldfish a few microvillous olfactory sensory neurons also show Gαi-3 or Gαq^5^, while in catfish, microvillous olfactory sensory neurons are associated with Gαq/11^4^. Furthermore, teleosts as well as cartilaginous fishes have a third class of olfactory sensory neurons, the crypt cells^8^,^9^,^10^. In teleosts, they carry the V1R type receptor ora4, associated with Gαi^11^ in zebrafish, but both Gαo and Gαq in goldfish^5^ and Gαo in catfish^4^. Within the teleostean olfactory epithelium, the cell somata of olfactory sensory neurons are typically positioned at different depths, with ciliated olfactory sensory neurons somata lying basally, crypt olfactory sensory neurons superficially and microvillous olfactory sensory neurons interspersed at intermediate depths. A recently discovered fourth olfactory sensory neuron type, the cap (kappe) cell, lodges also rather superficially in the zebrafish main olfactory epithelium; its receptor type is not known, but cap cells are associated with Gαo^12^. An overview of zebrafish olfactory receptor and G -protein classes in comparison to mouse is shown in Fig. 1B.

In the zebrafish, tracing of projections from olfactory sensory neurons using transgenic labelling of olfactory marker protein (OMP) visualized by RFP for ciliated olfactory sensory neurons and transient receptor potential channel 2 (TRPC2) visualized by Venus for microvillous olfactory sensory neurons^13^, as well as using calcium binding protein immunotracing^10^, indicate that ciliated olfactory sensory neurons terminate in anterior dorsal and ventromedial olfactory bulb glomeruli and that microvillous olfactory sensory neurons terminate in ventrolateral bulb glomeruli. Our own studies using calcium binding protein immunoreactivity^10^ showed furthermore that the posterior dorsomedial bulb area receives massive additional parvalbumin-positive projections from microvillous olfactory sensory neurons not visualized in the TRPC2-Venus transgenic line (and also not in the OMP-RFP line). Furthermore, crypt cell axons terminate in one single glomerulus within this mediodorsal area^9^,^10^,^14^. Secondary olfactory bulb projections, also using transgenic line labelling as well as tract tracing indicate some common projection targets of the entire olfactory bulb (posterior zone of dorsal telencephalon, ventral nucleus of ventral telencephalon), whereas more posterior ventral telencephalic areas (postcommissural nucleus of ventral telencephalon) and the habenula have been implied only to receive inputs from the mediodorsal area^15^,^16^.

The tetrapod amygdala is a complex structure composed of pallial and subpallial areas^17^. The medial amygdala is part of the subpallium and receives input from the tetrapod vomeronasal organ. Considerable progress has been made to differentiate between subpallial and pallial telencephalic areas in teleosts^18^,^19^,^20^,^21^ which is particularly critical for recognizing their relative contributions to the amygdala in teleosts. The teleostean medial zone of the dorsal telencephalon is generally considered to contain the homologue of the pallial amygdala^21^,^22^,^23^. There is also good evidence for recognizing in the zebrafish ventral telencephalic region (i.e. the subpallium) the septum (ventral nucleus of ventral telencephalon) and the basal ganglia (dorsal nucleus of ventral telencephalon)^24^. However, the more posterior subpallial nuclei, the supracommissural and postcommissural nuclei might correspond to the subpallial amygdala^24^. In the goldfish, but not in the zebrafish, an even more posterior subpallial region, the intermediate nucleus of ventral telencephalic area has been recognized as a separate olfactory bulb projection target in addition to other ventral telencephalic nuclei^25^. However, none of these posterior ventral telencephalic regions (postcommissural, supracommissural, intermediate nuclei of ventral telencephalon) has unequivocally been recognized as the teleostean medial amygdala.

The transcription factor Orthopedia (Otp) has various roles in zebrafish brain development. It is for example involved in the generation of neuropeptidergic cells of the supraopto-paraventricular region (SPV;^26^,^27^,^28^,^29^) and in the development of dopaminergic cells with long distance projections to spinal cord and to striatum in both mouse and zebrafish^30^,^31^. In embryonic mice, the SPV area seemingly contributes *otp-*expressing cells to the medial amygdala^32^. In accordance with their origin in the SPV, these cells are not GABAergic themselves, but become located in a principally GABA cell producing subpallial territory of the medial amygdala^33^,^34^. Similarly, in the zebrafish a dorsomedial stream of Otpa-positive cells (shown with immunohistochemistry) is seen to extend into a posterior domain of the telencephalon^29^ (Fig. 1A). If this Otpa-positive region receives secondary olfactory input, it might correspond to the intermediate nucleus of the ventral telencephalon described in the goldfish brain^25^ and, thus, represent the teleostean medial amygdala.

Zebrafish larvae imprint on visual and olfactory cues of their immediate kin (siblings from same batch) on day 5 and 6 postfertilization (dpf), respectively^35^,^36^,^37^ resulting in their ability to discriminate kin from non-kin later in life (kin recognition^37^). We use the term imprinting exclusively within this behavioral context. However, imprinting and resulting kin recognition does not occur in larvae that have experienced non-kin cues during the imprinting phase suggesting a predisposition for kin odor^38^ (see in this citation for details on the production of kin odor in our experiments). We previously analyzed which type(s) of olfactory sensory neuron(s) are activated by various olfactory stimuli using the activity marker phosphorylated Extracellular Signal Regulated Kinase (pERK) after stimulating imprinted zebrafish larvae with either non-kin conspecific odor or food odor^39^. While food odor activated both ciliated and microvillous olfactory sensory neurons, only the latter were activated by conspecific odor, but crypt cells showed no activation to both stimuli. Furthermore, tests with imprinted and non-imprinted zebrafish larvae (full siblings) for kin odor detection showed that crypt cells (and likely a subpopulation of microvillous olfactory sensory neurons, but not ciliated olfactory sensory neurons) were strongly activated only in imprinted fish and, thus, may play a role in detecting a kin odor related signal^39^. Thus, the sole olfactory bulb glomerulus mdG2 which receives crypt cell input and its likely next synaptic target, the postcommissural nucleus of the ventral telencephalon/intermediate nucleus of ventral telencephalon, might show enhanced or changed activity after kin odor stimulation.

In order to show that the posterior ventral telencephalic region called intermediate nucleus of ventral telencephalon corresponds to the zebrafish medial amygdala, we have used the following two approaches.

1. We investigated whether the known crypt cell projection target in the olfactory bulb (mdG2, identified with S100) and its likely next target, the intermediate nucleus of the ventral telencephalon (identified by Otpa) show increased or changed activity after kin odor stimulation in imprinted compared to non-imprinted zebrafish larvae by using an assay for pERK as performed successfully previously for the olfactory epithelium^39^.

2. We also performed tracing experiments in adult zebrafish to show the pathway from crypt cells via the mediodorsal olfactory bulb (incl. mdG2) into the Otpa-positive intermediate nucleus of the ventral telencephalic area, and from it to the tuberal hypothalamus, demonstrating an accessory olfactory system in teleosts.

In conclusion, we suggest that the intermediate nucleus of the ventral telencephalon is the homologue of the tetrapod medial amygdala based on its topology, transcription factor expression (Otpa), its neuronal circuitry (i.e. part of the accessory olfactory system) and, possibly, its changed activity after kin odor stimulation in non-imprinted larval fish (see Discussion).

## Results

### Neuroanatomical and neurochemical analysis

**Figure 1:**
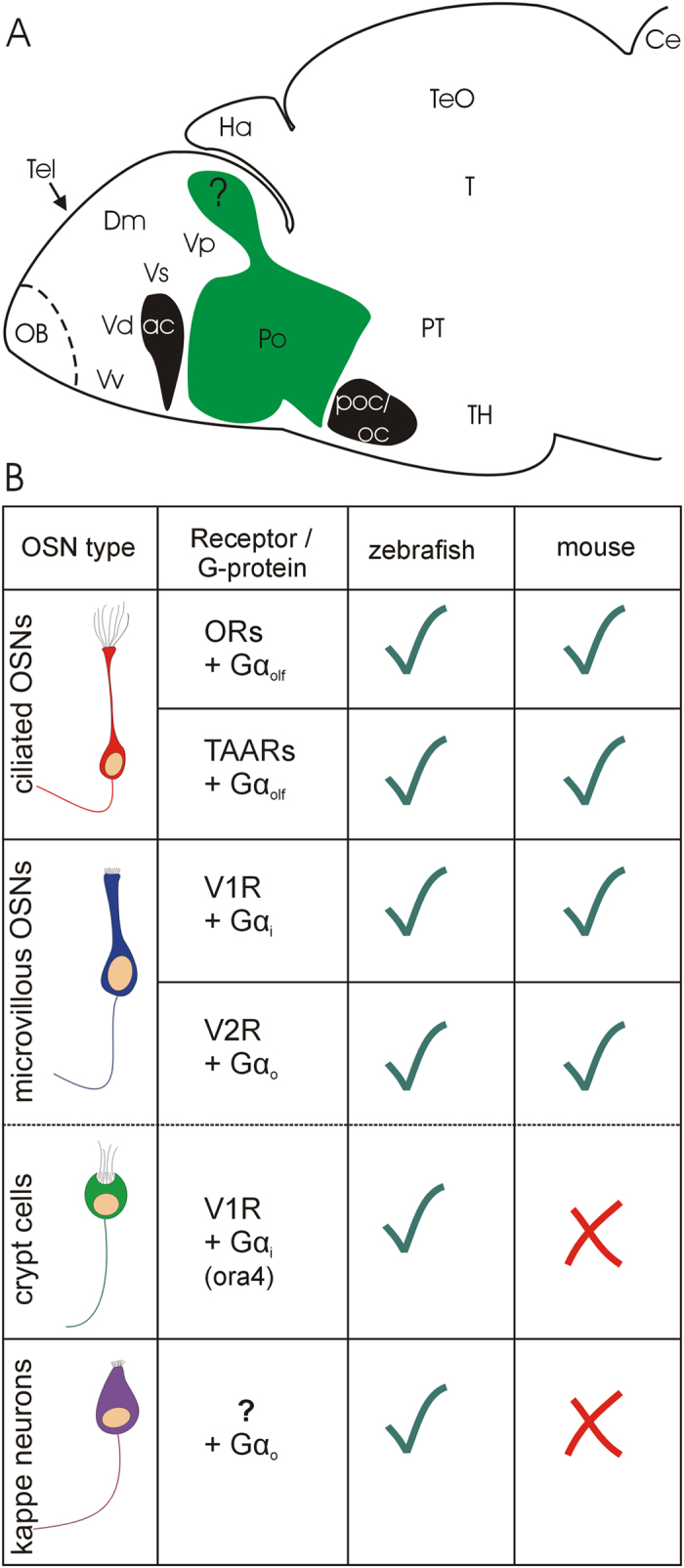
(**A**) The distribution of the transcription factor Otpa (shown in green) in the preoptic region and a posterior ventral telencephalic region (the latter indicated by a question mark) shown in a sagittal view of a 5 dpf zebrafish larva (drawn after^29^). Major telencephalic regions treated in the Introduction are schematically indicated. (**B**) Comparison of receptor molecules and associated G proteins on olfactory sensory neurons in zebrafish and mouse. See text for more details and references. Abbreviations: ac: anterior commissure; Ce: Cerebellum; Dm: medial zone of dorsal telencephalic area (pallium); Ha: Habenula; Po: preoptic region; poc/oc: postoptic commissure/optic chiasma; OB: olfactory bulb; PT: posterior tuberculum; T: midbrain tegmentum; TH: tuberal hypothalamus; Tel: telencephalon; TeO: tectum opticum; Vd, Vp, Vs; Vv: dorsal nucleus, postcommissural nucleus, supracommissural nucleus and ventral nucleus of ventral telencephalic area (subpallium).

**Figure 2:**
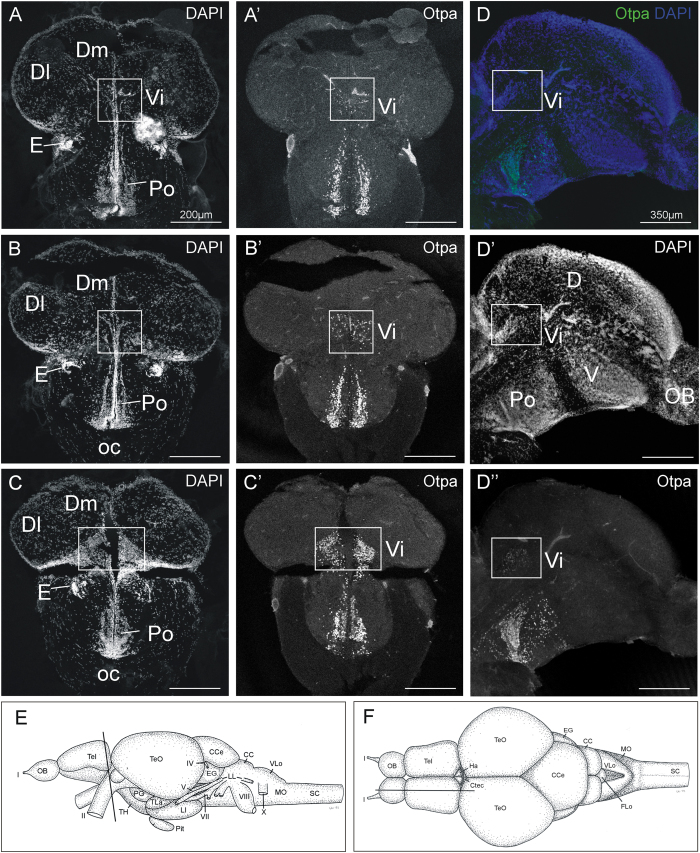
Neuroanatomical analysis and identification of the intermediate nucleus of ventral telencephalon using (**A–C,D,**D’) nuclear stain (DAPI) and (A’–C’,**D**,D”) immunohistochemistry for Otpa. (**E**) Lateral view of adult zebrafish brain shows level of sections shown in (**A-C,**A’–C’). (**F**) Dorsal view of adult zebrafish brain shows level of sections shown in (**D–**D”). Abbreviations: CC: crista cerebellaris; CCe: corpus cerebelli; Ctec: commissura tecti; D: dorsal telencephalic area; Dl: lateral zone of dorsal telencephalic area; Dm: medial zone of dorsal telencephalic area; EG: eminentia granularis; OB: olfactory bulb; LI: hypothalamic lobus inferior; LL: lateral line nerves; MO: medulla oblongata; MS: medulla spinalis; oc: optic chiasma; Pit: pituitary; Po: preoptic region; SC: spinal cord; TeO: optic tectum; TH: tuberal hypothalamus; TLa: torus lateralis; V: ventral telencephalon; Vi: intermediate nucleus of ventral telencephalon; VLo/LX: vagal lobe. I: olfactory nerve; II: optic nerve; IV: trochlear nerve; VII: facial nerve; VIII: octaval nerve; X: vagal nerve.

Sagittal sections of larval zebrafish immunostained for Otpa previously showed that Otpa-positive cells extend from the major Otpa expression domain in the neuroendocrine supraopto-paraventricular region into the posterior telencephalon (Fig. 1A; redrawn from data shown in ref. 29). This is the only Otpa-positive area in the entire telencephalon and is identified here as the intermediate nucleus of the ventral telencephalon which receives secondary olfactory (bulb) projections in the closely related goldfish^25^. Since a similar situation regarding *otp* expression and olfactory input has been reported in the mouse for the medial amygdala^32^,^33^,^34^, we undertook the tracing experiments in adult zebrafish brains in order to show that the intermediate nucleus of the ventral telencephalon receives secondary olfactory input. As will be reported below, in addition to the postcommissural nucleus of the ventral telencephalon, also the Otpa-positive intermediate nucleus of ventral telencephalon indeed receives secondary olfactory input from the mediodorsal olfactory bulb region which demonstrates that the intermediate nucleus of the ventral telencephalon qualifies as medial amygdala. The intermediate nucleus of the ventral telencephalon in the adult zebrafish brain lies in the extreme caudal pole of the telencephalon in the position where the telencephalon detaches from the preoptic region. A comparison of DAPI stains and Otpa immunostains visualizes these relationships nicely with various landmarks present at this level, such as the ventral entopeduncular nucleus (Fig. 2). Thus, both in sagittal (Fig. 2D–D”) as well as in transverse (Fig. 2A,A’’) adult zebrafish brain sections, the main well known Otpa expression domain known from the preoptic region of larval zebrafish^29^ can be seen to extend a thin stalk of Otpa-positive cells into the ventral telencephalic intermediate nucleus of the ventral telencephalon.

### Tracing experiments

#### Injections into dorsomedial olfactory bulb

**Figure 3:**
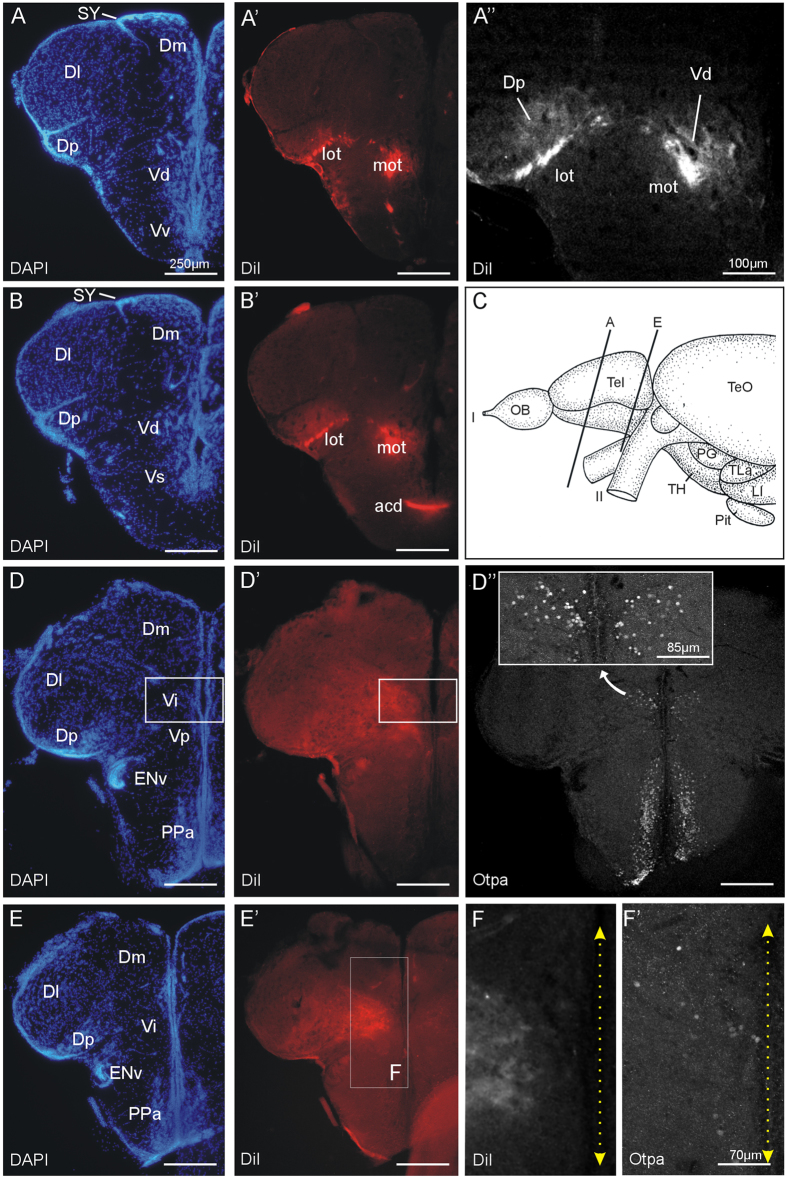
Projections after a unilateral DiI injection into the olfactory bulb of an adult zebrafish shown at four telencephalic levels from anterior (**A,**A’) to posterior (**E,**E’), with corresponding DAPI and fluorescent photomicrographs demonstrating tracing results. (A”) is a confocal blow-up at anterior levels detailing terminal fields and retrogradely labeled cells in the posterior zone of the dorsal telencephalon (Dp) and the dorsal nucleus of the ventral telencephalon (Vd). (**C**) shows section levels of (**A**) and (**E**). Sections (**B**) and (**D**) are the immediate caudal and rostral sections, respectively, and are not separately indicated. (D”) confocal photomicrograph shows Otpa staining in the preoptic region and the intermediate nucleus of the ventral telencephalon (Vi; insert). (**F,**F’) are confocal blow ups of E’ showing Di terminals and Otpa-positive cells in Vi. Stippled double arrows indicate midline. Abbreviations: acd: dorsal part of anterior commissure; Dm: medial zone of dorsal telencephalic area; Dl: lateral zone of dorsal telencephalic area; Dp: posterior zone of dorsal telencephalic area; OB: olfactory bulb; ENv: ventral entopeduncular nucleus; LI: hypothalamic lobus inferior; lot: lateral olfactory tract; mot: medial olfactory tract; PG: preglomerular complex; Pit: pituitary; Po: preoptic region; PPa: anterior parvocellular preoptic nucleus; TeO: optic tectum; TH: tuberal hypothalamus; TLa: torus lateralis; Vd: dorsal nucleus of ventral telencephalic area; SY: sulcus ypsiloniformis; Vi: intermediate nucleus of ventral telencephalon; Vp: posterior nucleus of ventral telencephalic area; Vv: ventral nucleus of ventral telencephalic area. I: olfactory nerve; II: optic nerve.

DiI injections into the adult zebrafish dorsomedial olfactory bulb results in massive labelling of medial and lateral olfactory tracts (mot, lot) and of secondary olfactory projections up to the caudal pole of the telencephalon (for an overview see Supplementary Figure S1). The lateral olfactory tract increasingly extends laterally posteriorly and underlies the posterior zone of the dorsal telencephalon issuing many small tracts and terminal fields in dorsal direction into this pallial zone from most anterior to most caudal telencephalic levels (Fig. 3A,A’–E,E’). Furthermore, retrogradely labelled cells are present in the posterior zone of the dorsal telencephalon and the dorsal nucleus of the ventral telencephalon which form the origin of a pallio-bulbar projection (Fig. 3A”). After entering the telencephalon, the medial olfactory tract initially runs laterally to the ventral nucleus of the ventral telencephalon and then remains lateral to the dorsal nucleus of the ventral telencephalon up to commissural levels (see Supplementary Figure S1). The medial olfactory tract may issue terminals to both the ventral and dorsal nuclei of the ventral telencephalon. At commissural levels, some fibers cross the midline via the anterior commissure (Fig. 3B’). At postcommissural levels the medial olfactory tract forms a large terminal field covering the postcommissural and intermediate nuclei of the ventral telencephalon (Vp, Vi; Fig. 3D,D’–E,E’). The intermediate nucleus of the ventral telencephalon is clearly identifiable at most caudal telencephalic levels where it is detected by Otpa immunohistochemistry subsequently performed on the same sections (3D”,F’). There is a dense terminal field of secondary olfactory bulb projections overlying these Otpa-positive cells. More laterally at these caudal levels, the Dp is still visibly covered by fine terminal fields coming from the lateral olfactory tract (Fig. 3E,E’).

#### Injections into tuberal hypothalamus

**Figure 4:**
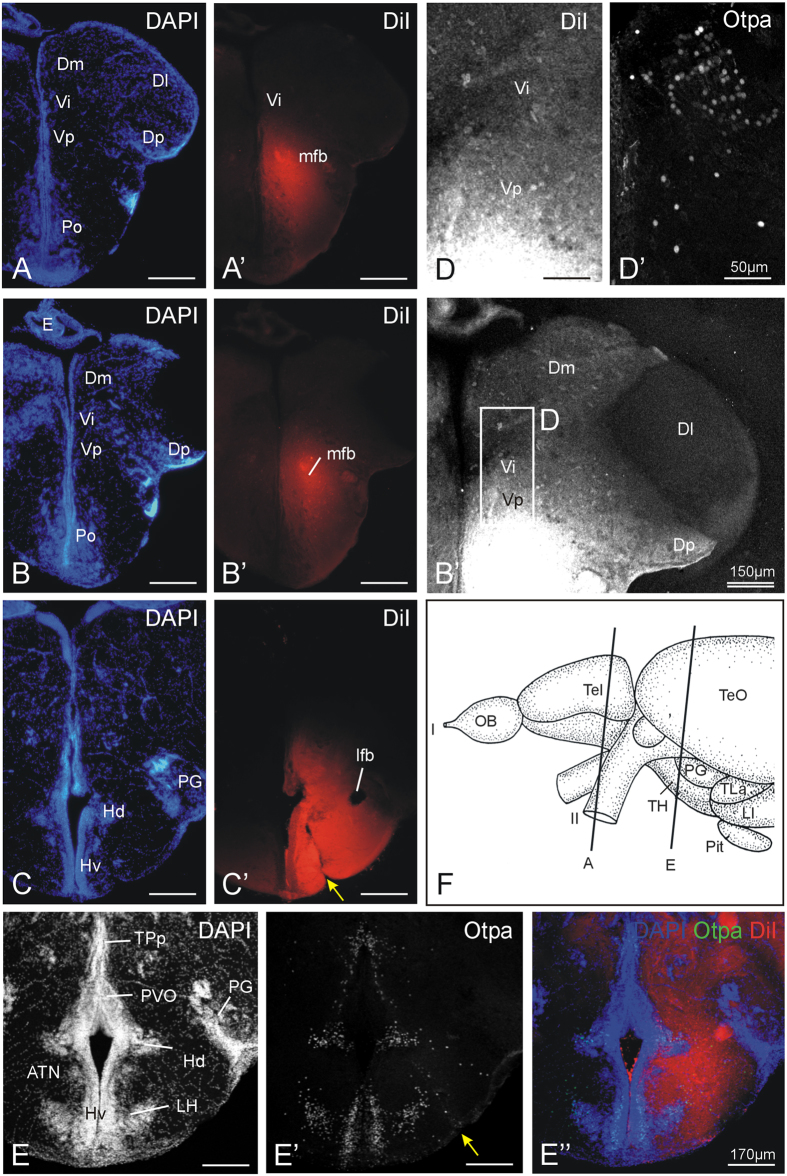
Neuronal connections after a unilateral DiI injection into the tuberal hypothalamus in adult zebrafish shown at three levels from anterior (**A,**A’) to posterior (**C,**C’; note yellow arrow at injection site) with corresponding DAPI and fluorescent photomicrographs demonstrating tracing results. (B”) Confocal photomicrograph shows retrograde tracing result in the telencephalon at the level of the intermediate nucleus of the ventral telencephalon (Vi). Note also that Dm, but not Dl, has retrogradely labeled cells (see text). (**D,**D’) shows confocal blow-up of B” (**D**) and corresponding Otpa stain (D’). (**E**–E”) details another injection site (yellow arrow) which is shown for DAPI, Otpa and DiI in confocal photomicrographs. (**F**) shows section levels of (**A**) and (**E**). Section (**B**) is immediately caudal to (**A**), and section (**C**) is at the same level as (**E**). Abbreviations: ATN: anterior tuberal nucleus; Dm: medial zone of dorsal telencephalic area; Dl: lateral zone of dorsal telencephalic area; Dp: posterior zone of dorsal telencephalic area; OB: olfactory bulb; E: epiphysis (pineal); ENv: ventral entopeduncular nucleus; Hd: dorsal zone of periventricular hypothalamus; Hv: ventral zone of periventricular hypothalamus; lfb: lateral forebrain bundle; LH: lateral hypothalamic nucleus; LI: hypothalamic lobus inferior; lot: lateral olfactory tract; mfb: medial forebrain bundle; mot: medial olfactory tract; PG: preglomerular complex; Pit: pituitary; Po: preoptic region; PPa: anterior parvocellular preoptic nucleus; PVO: paraventricular organ; Vd: dorsal nucleus of ventral telencephalic area; SY: sulcus ypsiloniformis; TeO: optic tectum; TH: tuberal hypothalamus; TLa: torus lateralis; TPp: periventricular part of posterior tuberculum; Vi: imtermediate nucleus of ventral telencephalon; Vp: posterior nucleus of ventral telencephalic area; Vv: ventral nucleus of ventral telencephalic area. I: olfactory nerve; II: optic nerve.

In order to show that the intermediate nucleus of the ventral telencephalon projects to the tuberal hypothalamus, we performed DiI injections into the area of the anterior tuberal nucleus and lateral hypothalamic nucleus. Because Otpa is also expressed in the tuberal hypothalamus (Fig. 4E,E’), i.e. in the ventral periventricular hypothalamic nucleus (Hv), as well as in the lateral hypothalamic nucleus (LH) and in the midline aspect of the periventricular nucleus of the dorsal hypothalamus (Hd) - but not in its major extent around the lateral hypothalamic recess - we can identify nicely the exact injection site (see two examples, Fig. 4C,C’). Many fibers affected by such injections can be traced in anterior direction running laterally to the preoptic area through the preoptic stalk into the telencephalon where they form part of the medial forebrain bundle. The lateral forebrain bundle remains unlabeled (Fig. 4C’) after tuberal hypothalamic injections. This fact fits well with anterograde tracing data in the goldfish where it was shown that the medial zone of the dorsal telencephalon (i.e. pallial amygdala homologue) projects via the medial forebrain bundle heavily into the tuberal hypothalamus whereas the lateral zone of the dorsal telencephalon (i.e. hippocampus homologue) projects via the lateral forebrain bundle to targets in the diencephalon other than the tuberal hypothalamus^40^.

In our experiments, the DiI labelled fibers can be followed into the postcommissural nucleus of the ventral telencephalon and the intermediate nucleus of the ventral telencephalon where many retrogradely labelled cell bodies are observed (Fig. 4B”). Interestingly within the pallium, retrogradely labelled cells are also seen in the medial zone of the dorsal telencephalon, but not in the lateral zone of the dorsal telencephalon (Fig. 4B”), which confirms the tracing data mentioned above in goldfish^40^. Immunostains for Otpa on the same DiI section demonstrate the presence of the Otpa-positive intermediate nucleus of the ventral telencephalon (Fig. 4D,D’). It is a hallmark of the mammalian accessory versus the main olfactory system that a part of the olfactory bulb (the accessory one) bypasses the olfactory cortex, but enters a subpallial amygdalar nucleus (the medial one) from which projections reach the tuberal hypothalamus in mammals^17^,^41^. Since our tracing experiments demonstrate a similar synaptic chain of connections we hypothesize the presence of an accessory olfactory system in teleosts as described in tetrapods leading sequentially via olfactory epithelium, dorsomedial olfactory bulb, and intermediate ventral telencephalic nucleus (i.e., medial amygdala) into tuberal hypothalamus (see Discussion for further reference).

### Kin odor stimulation experiment

**Figure 5:**
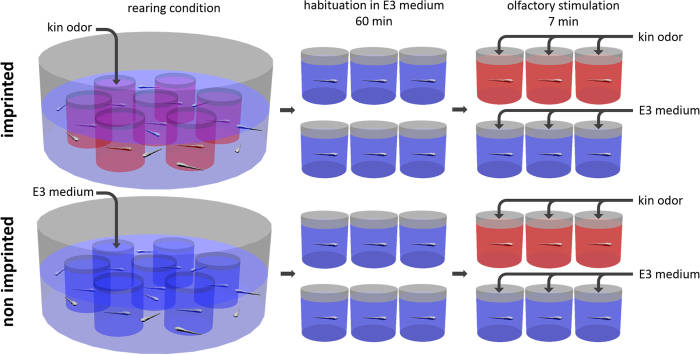
Experimental set-up: Schema shows how imprinted (red) and non-imprinted (blue) larval zebrafish were created and subsequently tested for kin odor activation. Zebrafish larvae were either exposed to kin odor or E3 medium at day 6 and both groups were subsequently tested either for kin odor or E3 medium at 9 days. Then, the larvae were sacrificed immediately after olfactory stimulation and further processed. Previous experiments had established that 7 minutes allow for optimal assay for pERK^39^. E3 medium is a commonly used medium for raising zebrafish eggs^49^.

**Figure 6:**
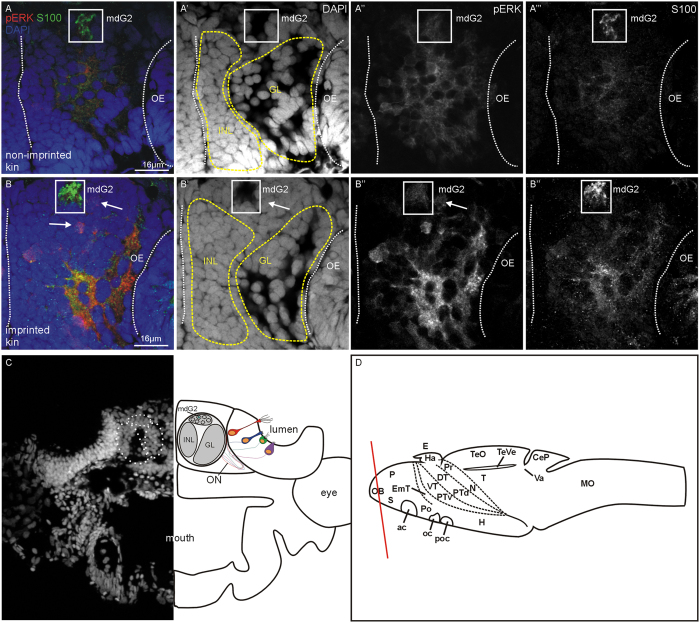
Example of pERK activation in olfactory bulb section containing mdG2 (frame) in imprinted and non-imprinted zebrafish larva. (**A**–A”’) confocal photomicrograph of a sectioned imprinted larva. (**B**–B”’) confocal photomicrograph of a sectioned non-imprinted larva. Channels comprise in addition to pERK, the nuclear stain DAPI and the calcium-binding protein immunostain S100. (**C**) Shows DAPI (left) and a schema with olfactory bulb fields that were counted (mdG2, INL, GL). (**D**) Larval brain in lateral view shows section level. Abbreviations: ac: anterior commissure; CeP: cerebellar plate; DT: dorsal thalamus (thalamus); E: epiphysis; EmT: eminentia thalami; GL: glomerular layer; H: hypothalamus; Ha: habenula; INL: inner nuclear layer; mdG2: mediodorsal glomerulus 2; MO: medulla oblongata; N: nucleus of the medial longitudinal fascicle; OB: olfactory bulb; ON: olfactory nerve; P: pallium; Po: preoptic region; Pr: pretectum; PTd, PTv: dorsal, ventral part of posterior tuberculum; S: subpallium; T: tegmentum; TeO: optic tectum; TeVe: tectal ventricle; Va: valvula cerebelli; VT: ventral thalamus (prethalamus).

**Figure 7:**
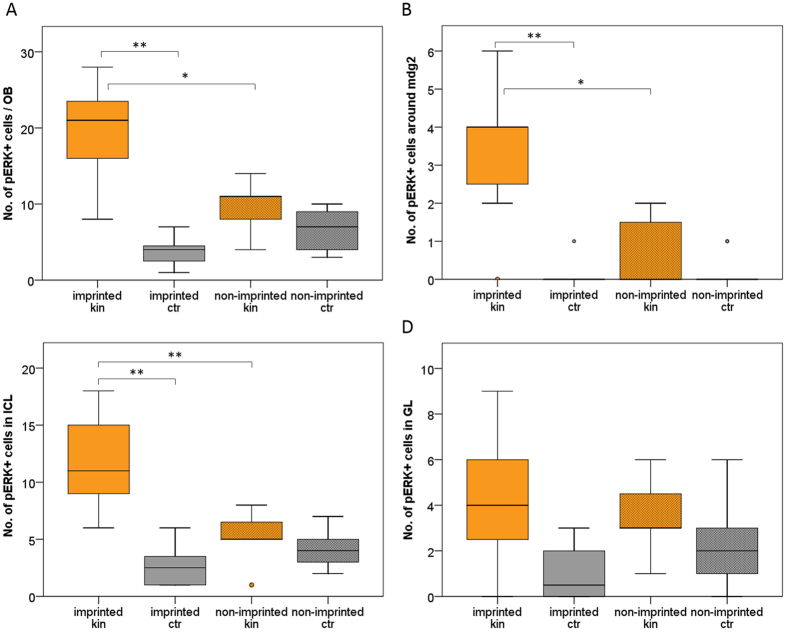
Number of pERK-positive cells in imprinted and non-imprinted zebrafish larvae, stimulated with either kin or control odor, in different olfactory bulb fields. Box plots show median (Mdn), upper and lower quartile and whiskers (maximum interquartile range: 1.5). *Indicates statistical significance p: *p < 0.05; **p < 0.01. kin = kin odor stimulus; ctr = control stimulus. n_imprinted kin_ = 7; n_imprinted ctr_ = 8; n_non-imprinted kin_ = 7; n_non-imprinted ctr_ = 9. (**A**) Total cell quantity of pERK-positive cells in entire olfactory bulb section at level of mdG2. Number of activated cells is significantly higher in imprinted larvae exposed to kin compared to imprinted larvae exposed to control stimulus (Mann-Whitney U = 6, p = 0.001, Mdn_impr kin_ = 21, Mdn_impr ctr_ = 4).Significant difference in cell number were detected between imprinted and non-imprinted larvae, exposed to kin (U = 6, p = 0.018, Mdn_impr kin_ = 21, Mdn_non-impr kin_ = 11). (**B**) pERK + cells around mdG2 (see Fig. 6) show a difference in activation between imprinted larvae stimulated with kin odor compared to imprinted larvae exposed to control stimuli (U = 4.5, p = 0.003, Mdn_impr kin_ = 4, Mdn_impr ctr_ = 0) or compared to non-imprinted larvae stimulated with kin odor (U = 6, p = 0.015, Mdn_impr kin_ = 4, Mdn_non-impr kin_ = 0). The same picture of neuronal activity arises in cells of inner cellular layer (**C**). Cell number differs significantly in imprinted larvae exposed to kin compared to imprinted larvae exposed to control stimulus (U = 0.5, p = 0.001, Mdn_impr kin_ = 11, Mdn_impr ctr_ = 3). pERK + cell number differs between imprinted larvae and non-imprinted larvae exposed to kin (U = 3, p = 0.006, Mdn_non-impr kin_ = 5). (**D**) shows the number of pERK + cells in glomerular layer of the olfactory bulb. A significant difference in pERK-positive cell number was found (Kruskall-Wallis test: H(2) = 9.357, p = 0.025); but after Mann-Whitney U correction for multiple comparisons (Bonferroni correction; α = 0.017) no significant differences could be detected between treatments for glomerular layer pERK + cell numbers (U = 9, p = 0.024, Mdn_impr kin_ = 4, Mdn_non-impr kin_ = 3).

**Figure 8:**
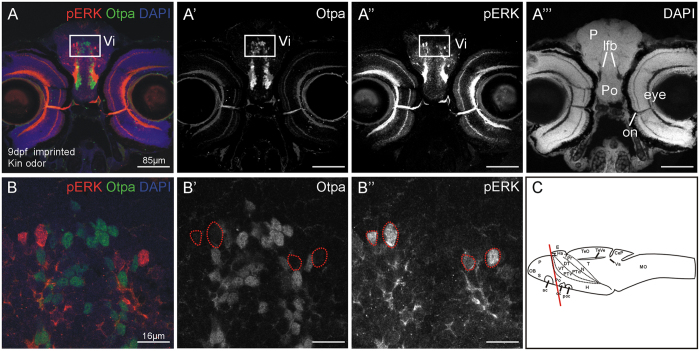
Example of pERK activation and Otpa-positive cells in the intermediate nucleus of the ventral telencephalon (Vi) and indication of the sector that was counted. (**A–A**”) Confocal photomicrographs show in addition to pERK, the nuclear stain DAPI and Otpa. (**B**) Higher power details of insert show pERK and Otpa-positive cells in confocal photography in histological material of tested fish. (**C**) Larval brain in lateral view shows section level. Abbreviations: ac: anterior commissure; CeP: cerebellar plate; DT: dorsal thalamus (thalamus); E: epiphysis; EmT: eminentia thalami; GL: glomerular layer; H: hypothalamus; Ha: habenula; INL: inner nuclear layer; lfb: lateral forebrain bundle; mdG2: mediodorsal glomerulus 2; MO: medulla oblongata; N: nucleus of the medial longitudinal fascicle; OB: olfactory bulb; on: optic nerve; P: pallium; Po: preoptic region; Pr: pretectum; PTd, PTv: dorsal, ventral part of posterior tuberculum; S: subpallium; T: tegmentum; TeO: optic tectum; TeVe: tectal ventricle; Va: valvula cerebelli; VT: ventral thalamus (prethalamus).

**Figure 9:**
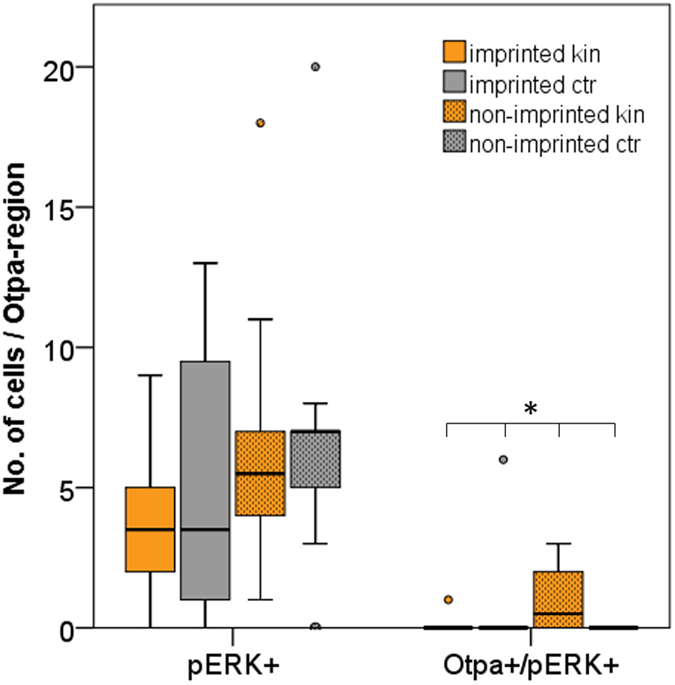
Analysis of pERK activated cell number in a restricted area of telencephalon, defined by Otpa staining. Both, pERK + cells, as well as double-labeled cells for Otpa and pERK were counted and analyzed in imprinted and non-imprinted zebrafish larvae, stimulated with either kin or control odor. Box plots show median (Mdn), upper and lower quartile and whiskers (maximum interquartile range: 1.5). *Indicates statistical significance p: *p < 0.05. kin = kin odor stimulus; ctr = control stimulus. n_imprinted kin_: 10; n_imprinted ctr_: 8; n_non-imprinted kin_: 10; n_non-imprinted ctr_: 9. Total cell quantity of single-labeled pERK-positive cells does not differ significantly between imprinted and non-imprinting larvae, stimulated with either kin odor or control stimulus (Kruskall-Wallis test: H(2) = 3.78, p = 0.295). Furthermore, pERK-positive and Otpa-positive double-labelled cell quantity was analyzed. A significant difference in cell number was found (H(2) = 8.579, p = 0.035) between treatments. A Mann-Whitney U test was performed to determine significant differences between two treatments. No significant differences could be detected after performing the Bonferroni correction (U = 28.5, p = 0.044, Mdn_non-impr kin_ = 0.5, Mdn_impr kin_ = 0; U = 22.5, p = 0.018, Mdn_non-impr kin_ = 0.5, Mdn_non-impr ctr_ = 0).

Two groups of wildtype zebrafish fertilized eggs (siblings) were raised in isolation in separate small glass beakers within two larger dishes each also containing their siblings (see Fig. 5). The latter later provided the visual kin signal to both groups of isolated fish. In contrast, the olfactory kin-related signals were applied during the critical time window individually to the isolated fish only in one group (the imprinted fish) whereas the other group (the non-imprinted fish) received a neutral signal (E3 water; see Fig. 5 and Methods for more details). This experiment has been reported before and demonstrated a role of crypt cells in the detection of kin specific odor by 9 day postfertilization (dpf) old larvae. This was shown through activation in olfactory sensory neurons using a pERK assay^39^. The difference between imprinted and non-imprinted fish was highly significant. However, neuronal activation in postsynaptic central nervous centers such as olfactory bulb and telencephalon had not been investigated in the previous study. Thus, we analyzed here whether there is a difference in activated neuronal cell numbers in the section containing the mdG2 glomerulus in imprinted and non-imprinted larvae (Figs 6 and 7). This section was visualized by using S100 immunohistochemistry which is only present in the olfactory bulb glomerulus mdG2 because all olfactory sensory neuron axons containing it converge there (Fig. 6; see also ref. 10). Similarly, we counted pERK-positive cells in a defined area in the telencephalon section containing the intermediate nucleus of the ventral telencephalon (Figs 8 and 9) which is visualized selectively by Otpa immunohistochemistry (see also previous paragraphs).

Already on first inspection, an olfactory bulb section taken from an imprinted larva tested with kin odor shows more pERK activated cells than the one from a non-imprinted larva tested in the same manner (compare Fig. 6A–A”’ and B–B”’). Cell counting included either all cells in this section or only those in the glomerular layer (GL), the inner cellular layer (ICL) or in the vicinity of mdG2 (Fig. 6A–D). A significant difference between imprinted and non-imprinted larvae (tested for kin odor) in pERK-positive cell numbers is seen for cells surrounding the mdG2 glomerulus and in the inner cellular layer (Fig. 7B–C). Furthermore, also the number of pERK-positive cells of entire sections differs significantly between imprinted and non-imprinted larvae (stimulated with kin odor) (Fig. 7A).

In addition, there is also a highly significant difference in activated cell numbers of imprinted fish tested with kin odor compared to the imprinted control fish (tested with E3 water) for total numbers of pERK bulb cells, as well as for the ICL and the area surrounding mdG2 (Fig. 7A–C). Due to the very conservative Bonferroni correction the significant difference in activated cells for the GL was discarded (for details see legend of Fig. 7D).

Next, activated cell numbers in a restricted telencephalic area defined by Otpa staining, i.e. the area of the intermediate nucleus of the ventral telencephalon (see Fig. 8), were counted, both for all activated (pERK-positive cells) as well as for cells double-labelled for Otpa and pERK (Fig. 9). A significant difference in cell numbers of double-labeled cells for Otpa and pERK between imprinted and non-imprinted larvae, stimulated with kin odor or control stimulus was found (Kruskall-Wallis Test; for details see legend of Fig. 9). The pairwise comparison of cell number revealed no differences in cell number due to a very conservative post hoc test (Mann-Whitney U test and Bonferroni correction; for details see legend of Fig. 9). However, the number of Otpa and pERK double positive cells tends to be increased in non-imprinted fish when tested for kin odor and compared to either control group or imprinted fish tested for kin (Fig. 9, right panel). All other comparisons did not yield differences for cell counts in this experiment.

Finally, we also tested 9 dpf old group-reared larvae taken from the same batch as the larvae used for the stimulation experiments in a 2-channel choice flume (as established in the Gerlach laboratory: see ref. 39) for successful imprinting.

## Discussion

The functional roles of teleostean ciliated and microvillous olfactory sensory neurons are somewhat puzzling. Ablation experiments in carp have associated ciliated cells with the alarm reaction response while food sensing has been correlated with microvillous olfactory sensory neurons, and crypt cells with reproduction^42^. Furthermore, while amino acids are sensed by both microvillous olfactory sensory neurons and ciliated olfactory sensory neurons, bile salts, gonadal steroids and prostaglandins are perceived by ciliated olfactory sensory neurons and nucleotides by microvillous olfactory sensory neurons^4^,^43^. This is somewhat unexpected because food signals (presumably indicated by nucleotids and amino acids) in mammals are typically sensed by ciliated olfactory sensory neurons.

The function of crypt cells has remained largely unknown (see Discussion in ref. 39). However, it had been established recently in the zebrafish that one receptor is present in all crypt cells (the V1R type receptor ora4 which is associated with Gαi;^11^) and that they project into the single S100-positive glomerulus located in the mediodorsal olfactory bulb, the mdG2^9^,^10^,^14^ (see Introduction). In addition, we have provided new data on neuronal activation using pERK suggesting a role of crypt cells in kin recognition^39^. These studies involved experimentally raised imprinted and non-imprinted zebrafish larvae (see Fig. 5), which were stimulated at 9 dpf with kin odor and immediately processed for visualization of pERK.

In the present contribution, we provide additional evidence that also the first central nervous processing station of crypt cell projections to the olfactory bulb glomerulus mdG2 is significantly elevated in activity as measured by the numbers of pERK-positive cells. In fact, comparing the section containing the S100-positive mdG2 glomerulus and counting all olfactory bulb cells in imprinted and non-imprinted 9 dpf larvae reveals that imprinted larvae have significantly more pERK activated cells compared to the imprinted control group (tested with E3 water; Fig. 7A). When certain compartments (see Fig. 6C for explanation of compartments) of the sections are analyzed, such as the inner cellular layer (ICL), the glomerular layer (GL), or the area surrounding mdG2, activated cell numbers are highly significantly increased in imprinted kin odor tested larvae versus controls in the area surrounding mdG2 (Fig. 7B) and in the ICL (Fig. 7C). Additionally, significant differences are also seen in activated, pERK-positive cell numbers of kin odor tested imprinted fish compared to non-imprinted kin odor tested fish in the ICL and in the area surrounding mdG2.

The results for the area of the intermediate nucleus of the ventral telencephalon are somewhat unexpected at first sight because no increase in the numbers of pERK activated cells (neither total pERK cells nor additionally Otpa-positive ones) after kin odor stimulation was seen in imprinted zebrafish larvae when compared to controls (Fig. 9, left panel). However, there was a significant difference in the number of activated cells positive for both Otpa and pERK. Cell numbers in **non**-imprinted fish are higher than in imprinted fish and control groups. This might indicate that non-imprinted fish show a neuronal response in the intermediate nucleus of the ventral telencephalon (the medial amygdala) to the unknown kin odor and that this response is alleviated or absent in larvae that had been imprinted. These activity differences in the zebrafish medial amygdala (intermediate nucleus of ventral telencephalon) in comparisons of imprinted and non-imprinted zebrafish larvae are highly exciting and clearly call for further investigation in particular with respect to additional inputs from microvillous cells. As reported before, in addition to the ubiquitous activation of crypt cells, a small subpopulation of microvillous olfactory sensory neurons was also activated by kin odor^39^.

These behavioral and neuronal activation experiments compare imprinted and non-imprinted zebrafish larva with respect to their response to kin odor and allow for tracking neuronal activation of the involved olfactory pathways from the sensory epithelium (crypt cells/some microvillous olfactory sensory neurons) central nervous system i.e. olfactory bulb (mdG2) and intermediate nucleus of the ventral telencephalon. Thus our results extend the functional neuroanatomical knowledge about the secondary olfactory circuitry of the mediodorsal olfactory bulb which includes the crypt cell target glomerulus mdG2^9^,^10^,^18^.

**Figure 10:**
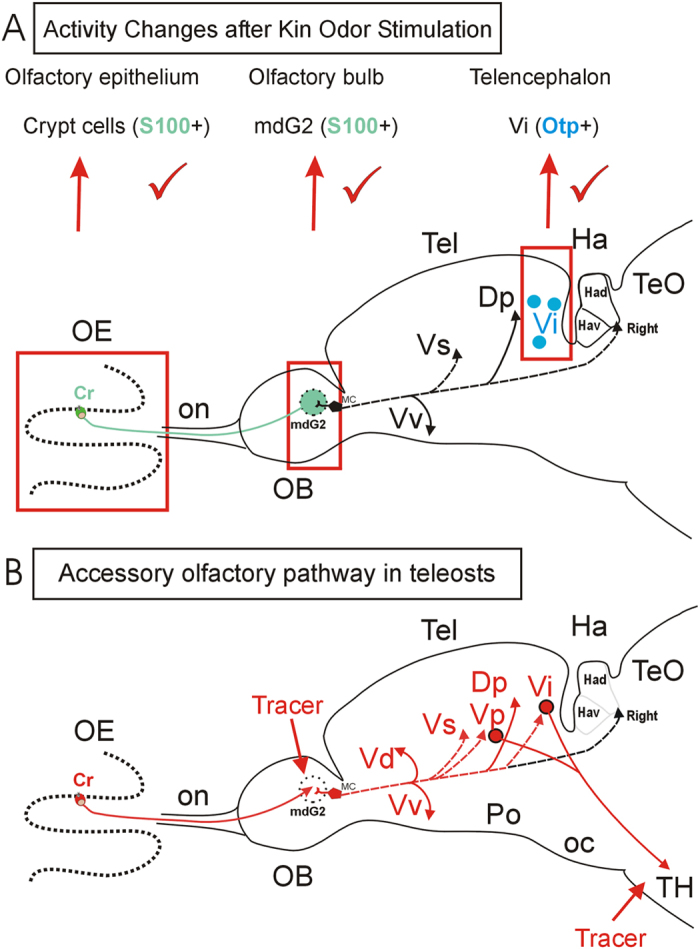
Schema of primary and secondary olfactory pathways in the adult zebrafish. (**A**) Neuronal activity quantified with pERK at three successive synaptic levels from peripheral sensory olfactory sensory neurons to central nervous targets (mdG2, which is immunohistochemically identified with S100 antibody because the projections of S100 immunopositive crypt cells terminate there; Vi, which is immunohistochemically identified with Otpa antibody for many of its cell bodies) after kin odor stimulation of imprinted and non-imprinted larvae. The counted pERK activated cells were located around the mdG2 and within Vi. Red tickmarks indicate significant changes in activated cell numbers seen at each level (see text for details). Higher order (i.e. secondary) olfactory projections of mediodorsal bulb area are indicated with solid black lines (targets shared with projections of entire olfactory bulb) and dashed black lines (targets specifically attributed to mediodorsal bulbar area; see literature below and text for more details). Crypt cells are widely distributed over the entire olfactory epithelium. (**B**) Projections of adult zebrafish mediodorsal olfactory bulb area (incl. mdG2) as shown in the present paper using the lipophilic tracing substance DiI (red lines). Tracer injections into tuberal hypothalamus (TH) demonstrate also a teleostean accessory olfactory pathway via Vp/Vi. Arrowheads indicate where a projection terminates. Olfactory bulb projections shown as dashed lines to telencephalic targets are selective for mediodorsal olfactory bulb (present paper and additional data from^9^,^10^,^13^,^14^,^15^,^16^,^44^). Abbreviations: Cr: crypt cells; Dp: posterior zone of dorsal telencephalon; Ha: habenula; Had: dorsal part of Ha; Hav: ventral part of Ha; OB: olfactory bulb; OE: olfactory epithelium; oc: optic chiasma; on: olfactory nerve; Po: preoptic region; Tel: telencephalon; TeO: optic tectum; TH: tuberal hypothalamus; Vd, Vp, Vs, Vv: dorsal, postcommissural, supracommissural, ventral nucleus of ventral telencephalon.

Previous studies had already shown that the zebrafish mediodorsal olfactory bulb receives preferential input from microvillous cells and projects – in addition to olfactory bulb targets common to the entire olfactory bulb (posterior zone of dorsal telencephalon, ventral nucleus of ventral telencephalon) – also to the postcommissural and supracommissural ventral telencephalic nuclei, as well as to the right dorsal habenula (Fig. 10; Vp, Vs, Had;^10^,^16^,^44^). Our tracing data presented here demonstrate that the adult zebrafish mediodorsal olfactory bulb area not only projects to the posterior zone of the dorsal telencephalon, as well as to the dorsal, ventral, supracommissural and postcommissural nuclei of the ventral telencephalon, but in addition also to the intermediate nucleus of the ventral telencephalon. We are certain about this projection to intermediate nucleus of the ventral telencephalon because our DiI tracing experiments involved the parallel immunohistochemical visualization of the Otpa-positive cells in the intermediate nucleus of the ventral telencephalon (Fig. 3D-D”) which is diagnostic for this nucleus. Since *otp* is also solely expressed in the mammalian medial amygdala within the telencephalon (see Introduction), we confidently identify the intermediate nucleus of the ventral telencephalon as teleostean medial amygdala. This is further supported by the fact that the intermediate nucleus of the ventral telencephalon projects to the tuberal hypothalamus, more specifically, the region of the ventral periventricular hypothalamic zone (Hv), and the anterior tuberal nucleus and lateral hypothalamic nucleus where our DiI injections were located (Fig. 4C,C,E-E”). The zebrafish Hv has previously been identified as the homologue of the mammalian hypothalamic arcuate nucleus^45^.

Such higher order olfactory circuitry is diagnostic in mammals for the accessory olfactory system originating there in microvillous olfactory sensory neurons of the vomeronasal organ and running via the accessory olfactory tract and bulb into the subpallial medial amygdala and from there directly into the tuberal hypothalamus^41^ unlike the targets of the main olfactory pathway(s). In fact, a similar situation exists for all tetrapods^17^. What is more, a previous paper in the African lungfish *Protopterus* has shown that a “hidden” accessory olfactory system exists in this basal sarcopterygian clade. Lungfish, like teleosts, have no vomeronasal organ separate from the main olfactory epithelium^46^. However, similar to teleosts, lungfish also have a specialized circuit originating from microvillous “crypts” (containing microvillous cells, not to be confounded with teleostean crypt cells) in the main olfactory sensory epithelium via a special part of the olfactory bulb to medial amygdala and from there to tuberal hypothalamus^46^. This shows, together with our results presented here in the zebrafish, that a dichotomous presence of the main and accessory olfactory system is basal to all bony vertebrates. The condition in cartilaginous fishes remains to be established. However, a possible dichotomy of the olfactory system has recently been reported in agnathans^47^,^48^, although the accessory olfactory system in lampreys may be analogous rather than homologous to the vomeronasal system because it runs from the olfactory bulb directly via the basal diencephalic posterior tuberculum into the rhombencephalon

Thus, the evidence for identifying the intermediate nucleus of the ventral telencephalon as the medial amygdala of teleost fishes is based on the following findings: (A) the intermediate nucleus of the ventral telencephalon lies topologically between pallial amygdala (medial zone of dorsal telencephalon) and the remainder of the subpallial amygdala (supracommissural nucleus of ventral telencephalon, postcommissural nucleus of ventral telencephalon; see Fig. 2). (B) The intermediate nucleus of the ventral telencephalon is the sole expression domain of Otpa in the telencephalon (see Fig. 2). (C) The intermediate nucleus of the ventral telencephalon receives mediodorsal olfactory bulb projections shown with anterograde tracing (see Fig. 3). (D) The intermediate nucleus of the ventral telencephalon projects to the tuberal hypothalamus as shown with retrograde tracing (see Fig. 4). (E) There is phylogenetic continuity (teleosts, lungfish, amphibians, and amniotes) indicating that a medial amygdala exists before the divergence of actinopterygian and sarcopterygian fishes.

In summary, this study shows that imprinted and non-imprinted larval zebrafish differ in neuronal activation after kin odor exposure in three successive synaptic levels along the pathway leading from olfactory epithelium to telencephalon (Fig. 10A). Our tracing experiments furthermore show that an accessory olfactory system indeed does exist in teleosts which originates in crypt and microvillous olfactory sensory cells and runs sequentially via the dorsomedial olfactory bulb and medial amygdala (intermediate nucleus of ventral telencephalon) to the tuberal hypothalamus (Fig. 10B).

## Methods

The experimental set-up for creating imprinted and non-imprinted zebrafish larvae and subsequent testing with kin odor (see Fig. 5), followed by histological processing for detection of neuronal activation has been described in detail already^39^. Briefly, we recapitulate these issues below. The tracing study involved different adult zebrafish specimens and the histological processing for visualizing the neuronal tracer as well as the concurrent immunohistological identification of the transcription factor Otpa.

### Study animals and rearing conditions

Adult zebrafish wildtype used in Oldenburg were obtained from different commercial breeding facilities (Germany, Vietnam, Sri Lanka) and maintained in 3 liter aquaria per breeding pair at 26 °C under a 13 h:11 h light:dark cycle. Fish were fed daily, alternating with commercial flake food, *Artemia salina* and white mosquito larvae. For breeding spawning trays were used. Eggs were kept in E3 medium^49^ in an incubator at the same temperature and light conditions as the adults. Larvae hatched at 3–4 day post fertilization (dpf). After depletion of the yolk (on 5 dpf) larvae were fed with commercial fry food and *Paramecium spec*. Eggs and larvae were reared according to kin odor stimulation experiment conditions (see Fig. 5).

Animal Use and Care Protocols were approved by the Institutional Animal Care and Use Committee of the University of Oldenburg and the government of the state Niedersachsen, Germany (18.01.2013–17.01.2016). All experiments were carried out in accordance with the approved guidelines. After the experiment, larvae were killed by an overdose of MS222 (Sigma-Aldrich).

Adult zebrafish wildtype used in Munich for immunohistochemical and tracing experiments came from the zebrafish facility at the Ludwig-Maximilians-University (LMU) Munich. Zebrafish were kept in a zebrafish housing system (ZebTEC,stand-alone-unit,Tecniplast©) at a temperature of 28 °C and a 12/12 light/dark cycle. Animals used in the tracing study were treated according to the German regulations on Animal Protection (Deutsches Tierschutzgesetz). Tracing experiments conducted in this paper involved no animal experiments in the sense of the German Animal Protection law. We used fixed brain tissue from decapitated adult zebrafish that were killed with an overdose of MS222 (Sigma-Aldrich).

### Odor choice test

As described in our previous paper, successful olfactory preference tests were performed on imprinted full siblings of those 9 dpf larvae which were stimulated with kin odor and analyzed for upregulation of pERK in the olfactory epithelium^39^ and in the central nervous system (present contribution; see Figs 5).

### Stimulation experiments with kin odor

Imprinted and non-imprinted larvae of the same batch were reared and treated according to our previous study regarding neuronal activation in the olfactory epithelium after kin odor exposure^39^.

### Tracing Experiments

Adult zebrafish (n = 9) were deeply anesthetized with MS222 (Sigma, Aldrich) before decapitation and fixation of the brains for 48 h in 4% paraformaldehyde (PFA) in Sörensen phosphate buffer (PB). Fine crystals of DiI (D3911; Molecular Probes) were applied with a fine needle to the dorsomedial area of the olfactory bulb or the tuberal hypothalamus unilaterally and the injection site was sealed with warm Agar-agar (4% in Aq. dest). After 5 to 6 days of incubation in PFA-PB fixative in an incubation chamber at 37 °C, the brains were rinsed in PB and embedded in Agar-agar (4% in Aq. dest) and cut at 30–50 μm on a vibratome (Leica, VT1000 S). Sections were collected on Superfrost Plus glass slides (Thermo Scientific) counterstained with DAPI (40–6-diamidino-2-phenylindole; 1:1000 dilution, Carl Roth), mounted with Vectashield (Vectorlabs) and coverslipped. DiI positive neuronal connections were then photographed (see below) before coverslips were removed and sections further processed for Otpa immunohistochemistry (see next section).

### Tissue preparation and immunohistochemical processing

Staining for pERK/S100: imprinted and non-imprinted larvae were processed according to our previous study^39^.

Staining for Otpa: Rat polyclonal antibodies against the transcription factor Otpa (Immunogen: Synthetic peptide CKKPVHPGDLAPVSDA) were manufactured by Covance (USA) and used on our brain tissue at a dilution of 1:500. The specificity of this custom-made antibody was confirmed in 3dpf *otpa*^m866^ mutant larvae^30^ and in 5dpf *otpa*^m866^ mutant larvae^29^.

Cryostate sections of imprinted and non-imprinted larvae were additionally incubated with a third primary antibody against Otpa (rat anti Otpa, dilution 1:500) diluted in blocking solution for 2 days at 4 °C in a humid chamber. Afterwards slides were washed with PBS and incubated with the third secondary antibody (AMCA, anti rat, Dianova or 488 anti rat, Dianova, 1:300,) diluted in blocking solution for 3 hours in a humid chamber at room temperature.

Coverslip of adult zebrafish brain vibratome (DiI) sections were washed off for final Otpa immunohistochemical processing in PBS. Additional adult zebrafish brains were cryosectioned at 30 μm for neurochemical/-anatomical analysis. Sections were incubated with proteinase-K (Sigma, P6556, 1:1000, diluted in PBS) for 15 min at 37 °C to facilitate antibody penetration. Afterwards, all adult sections were processed for Otpa immunostaining as described for larval sections above.

### Confocal microscopy

Optical larval and adult tissue sections were acquired with a Leica TCS SP-5 confocal laser-scanning microscope (Leica Microsystems). All microscopic images used in this study were processed to RGB stacks and projections by using ImageJ and slightly adapted for brightness and contrast with either ImageJ or Corel PHOTO-PAINT 12.0. Photographic plates were mounted and further processed into figures with CorelDRAW 12.0 (Corel Corporation).

### Epifluorescence microscopy

Photomicrographs of sectioned adult brain tissue were taken with a light/fluorescence microscope (Nikon Eclipse 80i; Nikon Instruments Inc.) equipped with Nikon Plan Fluor 109/0.30 (10x) and Plan Fluor 209/0.50 (20x) objectives, a Nikon Digital Sight DSU1 Photomicrographic Camera (Nikon Instruments Inc.) and LUCIA-G5 software.

### Quantification of activated cells

Stacks of olfactory bulb sections were analyzed by using the RoiManager tool of ImageJ and all activated cells (pERK+) were counted. Position of cell-soma was identified according to assignment to one of three areas shown in Fig. 6C. Appropriate section was identified with immunohistochemistry for the calcium binding protein S100 which labels the neuropil in the center of the mdG2 glomerulus (as described previously^10^).

Similarly, telencephalon sections containing the intermediate nucleus of the ventral telencephalon (Vi, identified with immunohistochemistry for Otpa) were analyzed in the same way for pERK-positive cells and additionally for pERK/Otpa-positive cells in a defined sector (see Fig. 8).

Cell counting for statistical analysis was performed blind, by two observers unknowingly which specimen (imprinted/non-imprinted) and stimulus (control/kin odor) they were evaluating.

### Statistical evaluation

After kin odor tests, the quantity of pERK-positive cells was counted in the section with the S100-positive mdG2 glomerulus in the total olfactory bulb (Fig. 7A), the inner cellular layer (B), in the glomerular layer (C) or in the area surrounding the mdG2 (D; see also Fig. 6C for explanation) both in imprinted and non-imprinted larvae. Furthermore, pERK-positive labeled cells and Otpa-positive/pERK-positive double labeled cells were quantified in a defined telencephalic field containing the Otpa-positive intermediate ventral telencephalic nucleus (Vi) in imprinted and non-imprinted larvae, either stimulated with kin or control stimulus.,

Cell quantity was analyzed using a nonparametric Kruskall-Wallis test followed by a pairwise Mann-Whitney U test including Bonferroni correction (α = 0.017). All analyses are two-tailed and were done in IBM SPSS statistic 23 for windows.

## Additional Information

**How to cite this article:** Biechl, D. *et al*. Identification of accessory olfactory system and medial amygdala in the zebrafish. *Sci. Rep.* **7**, 44295; doi: 10.1038/srep44295 (2017).

**Publisher's note:** Springer Nature remains neutral with regard to jurisdictional claims in published maps and institutional affiliations.

## Supplementary Material

Supplementary Information
