# Supplementary material for: Identification of accessory olfactory system and medial amygdala in the zebrafish
Source: Sci Rep. 2017 Mar 14;7:44295. doi: 10.1038/srep44295 (PMC5349599; doi:10.1038/srep44295)
Supplement: Supplementary Information [file srep44295-s1.pdf]

Supplementary Figure 1

Identification of accessory olfactory system and medial amygdala in the zebrafish

Daniela Biechl, Kristin Tietje, Soojin Ryu, Benedikt Grothe, Gabriele Gerlach,

Mario F Wullimann

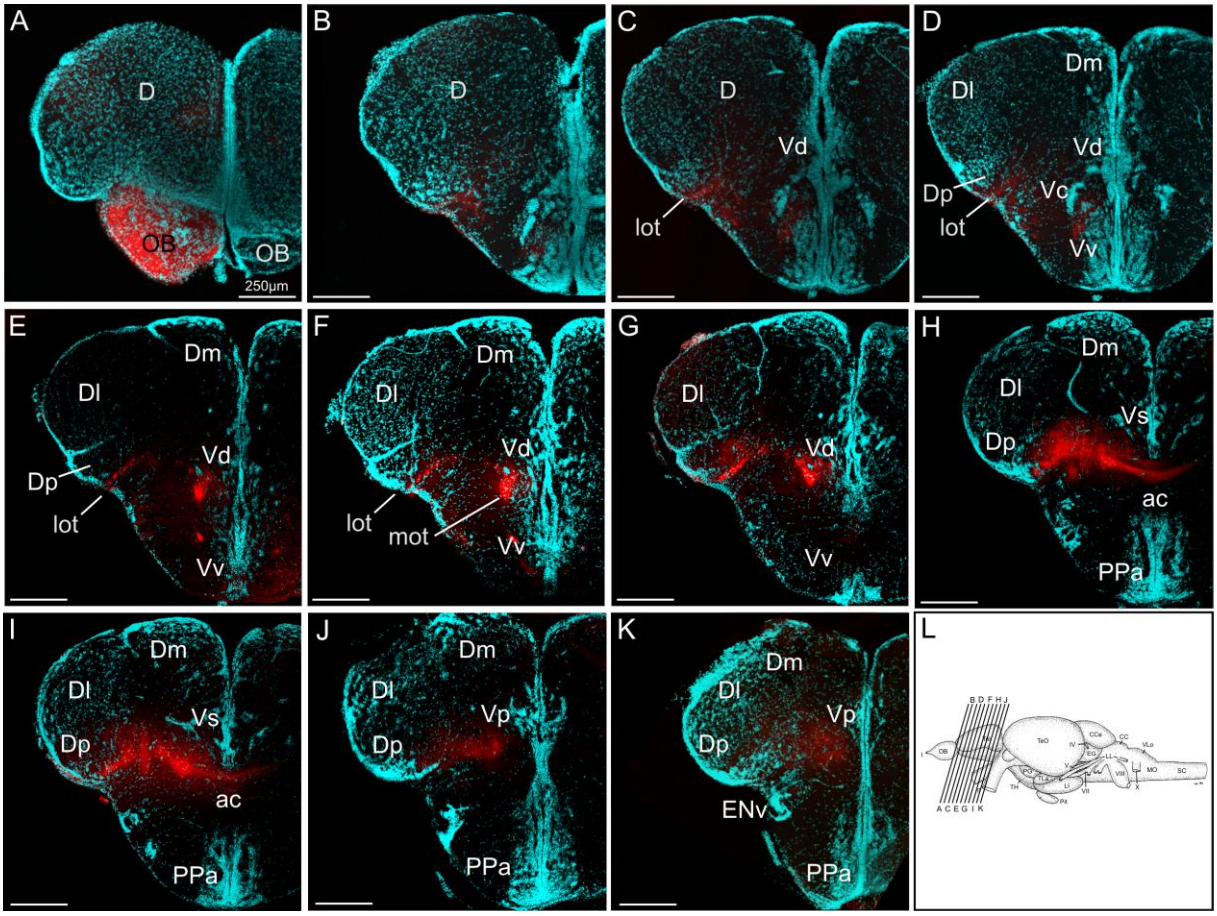

Supplementary Figure 1: **Olfactory bulb projections in adult zebrafish** (A) – (K) Series of cross-sections through an adult zebrafish telencephalon showing secondary olfactory projections following an olfactory bulb DiI tracer injection. (L) Levels of sections. For abbreviations: see main paper.
